# Applying Wearable Sensors and Machine Learning to the Diagnostic Challenge of Distinguishing Parkinson’s Disease from Other Forms of Parkinsonism

**DOI:** 10.3390/biomedicines13030572

**Published:** 2025-02-25

**Authors:** Rana M. Khalil, Lisa M. Shulman, Ann L. Gruber-Baldini, Stephen G. Reich, Joseph M. Savitt, Jeffrey M. Hausdorff, Rainer von Coelln, Michael P. Cummings

**Affiliations:** 1 Center for Bioinformatics and Computational Biology, University of Maryland, College Park, MD 20742, USA; rmkhalil@umd.edu; 2 Department of Neurology, University of Maryland School of Medicine, Baltimore, MD 21201, USA; lshulman@som.umaryland.edu (L.M.S.); sreich@som.umaryland.edu (S.G.R.); jsavitt@som.umaryland.edu (J.M.S.); 3 Department of Epidemiology and Public Health, University of Maryland School of Medicine, Baltimore, MD 21201, USA; abaldin@som.umaryland.edu; 4 Center for the Study of Movement, Cognition, and Mobility, Neurological Institute, Tel Aviv Medical Center, Tel Aviv 6492416, Israel; jeffh@tlvmc.gov.il; 5 Department of Physical Therapy, Faculty of Medicine & Health Sciences, Tel Aviv University, Tel Aviv 6997801, Israel; 6 Sagol School of Neuroscience, Tel Aviv University, Tel Aviv 6997801, Israel; 7 Rush Alzheimer’s Disease Center, Rush University Medical Center, Chicago, IL 60612, USA; 8 Department of Orthopedic Surgery, Rush University Medical Center, Chicago, IL 60612, USA

**Keywords:** Parkinson’s disease, parkinsonism, neurodegenerative diseases, machine learning, wearable sensors, mobility testing, diagnosis, timed up and go test

## Abstract

**Background/Objectives:** Parkinson’s Disease (PD) and other forms of parkinsonism share motor symptoms, including tremor, bradykinesia, and rigidity. The overlap in their clinical presentation creates a diagnostic challenge, as conventional methods rely heavily on clinical expertise, which can be subjective and inconsistent. This highlights the need for objective, data-driven approaches such as machine learning (ML) in this area. However, applying ML to clinical datasets faces challenges such as imbalanced class distributions, small sample sizes for non-PD parkinsonism, and heterogeneity within the non-PD group. **Methods:** This study analyzed wearable sensor data from 260 PD participants and 18 individuals with etiologically diverse forms of non-PD parkinsonism, which were collected during clinical mobility tasks using a single sensor placed on the lower back. We evaluated the performance of ML models in distinguishing these two groups and identified the most informative mobility tasks for classification. Additionally, we examined the clinical characteristics of misclassified participants and presented case studies of common challenges in clinical practice, including diagnostic uncertainty at the patient’s initial visit and changes in diagnosis over time. We also suggested potential steps to address the dataset challenges which limited the models’ performance. **Results:** Feature importance analysis revealed the Timed Up and Go (TUG) task as the most informative for classification. When using the TUG test alone, the models’ performance exceeded that of combining all tasks, achieving a balanced accuracy of 78.2%, which is within 0.2% of the balanced diagnostic accuracy of movement disorder experts. We also identified differences in some clinical scores between the participants correctly and falsely classified by our models. **Conclusions:** These findings demonstrate the feasibility of using ML and wearable sensors for differentiating PD from other parkinsonian disorders, addressing key challenges in its diagnosis and streamlining diagnostic workflows.

## 1. Introduction

Distinguishing Parkinson’s disease (PD) from other forms of parkinsonism poses a significant clinical challenge due to overlapping clinical characteristics among the different disease entities and the marked heterogeneity in the clinical presentation of each form of parkinsonism, including in idiopathic PD itself [1]. In addition to PD, parkinsonism can be the core manifestation of other neurodegenerative diseases, including multiple system atrophy (MSA), progressive supranuclear palsy (PSP), corticobasal syndrome (CBS), and dementia with Lewy bodies (DLB), and secondary forms of parkinsonism, such as drug-induced parkinsonism (DIP) and vascular parkinsonism. Furthermore, other movement disorders (especially essential tremor (ET)) can be associated with relatively mild parkinsonism over time [2]. Some ET patients exhibit more complex phenotypes (including parkinsonian signs), while others may eventually progress to PD, adding to the challenge of making a diagnosis [2]. Each of these conditions is characterized by distinct neuropathological and phenotypic features [3]. However, they all share some degree of the motor symptoms typical of PD, including tremor, bradykinesia, and rigidity, although with varying prominence [4]. Additionally, mild parkinsonism without concomitant PD can develop with aging, further complicating an accurate diagnosis [5]. The lack of definitive biomarkers and the reliance on clinical criteria for diagnosis contribute to diagnostic uncertainty, often leading to misclassification, especially early in the disease, when the signs and symptoms of different forms of parkinsonism have greater overlap and distinguishing features can be subtle or absent [6,7].

Diagnostic accuracy in the clinical diagnosis of PD is highly dependent on the experience and expertise of the treating clinician. A recent comprehensive meta-analysis revealed a balanced diagnostic accuracy ranging from 69.5% for non-experts to 82.4% for movement disorder experts [8]. In other words, up to 26% of patients were initially misdiagnosed, based on the clinical diagnostic criteria of PD [8]. The most common misdiagnoses were due to the significant clinical similarities between PD and other neurodegenerative forms of parkinsonism. The diagnostic accuracy for other parkinsonian disorders is even lower than that of PD [9]. We recently showed that the movement disorder specialists at our own center changed their clinical diagnosis of PD in 6% of patients over a 15-year period [10]. The most frequent diagnostic changes involving PD were observed with DIP, MSA, PSP, and DLB. Treatment and prognoses differ considerably between these different underlying conditions [3]. Some additional tools commonly used in clinical practice, such as imaging tests (e.g., DaTscan, MIBG scintigraphy), can assist clinicians in differentiating PD from other parkinsonian syndromes by introducing greater objectivity into the diagnostic process [11]. Nonetheless, despite these advancements, the final diagnosis remains largely dependent on clinical examination. Consequently, the development of objective diagnostic tools capable of distinguishing PD from other forms of parkinsonism remains a critical unmet need in both clinical practice and research.

Wearable sensors containing accelerometers and gyroscopes provide continuous, highly granular, and objective measurements that can be used to identify subtle changes in complex movement patterns [12,13,14], even in an early stage of the disease when abnormalities may be too subtle to be detected by clinical assessment alone [15]. Although statistical analysis of wearable sensor data has shown promise in the differential diagnosis of parkinsonian disorders [16,17,18,19,20], ML provides a more advanced and adaptable approach for analyzing the large-scale, high-dimensional, and complex datasets generated by these sensors. ML analyses can identify subtle patterns and underlying relationships that may not be detectable through conventional statistical methods or clinical observation [21,22]. By analyzing sensor-derived movement data from individuals with PD and various parkinsonian syndromes, ML models can extract distinctive kinematic signatures and biomarkers associated with specific parkinsonian syndromes [23]. Previous studies have used ML to distinguish PD from other forms of parkinsonism, demonstrating its potential to support clinical decision-making [24,25,26,27,28]. The ability to classify PD from other types of parkinsonism is clinically relevant in terms of patient/family counseling, the prediction of disease progression, and determining the most appropriate treatment strategies [29,30].

We conducted a search of the literature using the PubMed, IEEE Xplore, and Nature portofolio databases, covering publications from 1995 to 2024. The search keywords included “((artificial intelligence) OR (machine learning) OR (deep learning)) AND (Parkinson) AND ((atypical parkinsonism) OR (differential diagnoses)) AND (wearable OR gait)”. We found that previous research applying ML focused predominantly on distinguishing PD from a single type of parkinsonism [26,27,28,31,32,33,34,35]. Only one study applied ML to a smartwatch dataset to differentiate PD from various differential diagnoses, including ET, atypical parkinsonism such as PSP or MSA, secondary causes of parkinsonism, and Multiple Sclerosis [24]. Another study developed an ML system to differentiate between PD, cerebellar ataxia, and PSP-Richardson syndrome (PSP-RS) based on gait and postural instability data [25]. However, this approach involved the construction of two separate binary classifiers: one distinguishing PD from ataxia and another distinguishing PD from PSP-RS.

Using ML to distinguish participants with PD from a diverse group of types of parkinsonism in real-world clinical datasets poses significant challenges. First, the overlapping motor symptoms shared by both groups complicate the ability of ML models to accurately distinguish between them. Second, the non-PD parkinsonism groups generally have a much smaller sample size compared to the PD group, as PD accounts for more than 70% of all cases of parkinsonism [36]. Studies conducted in various countries have shown that PD is the most frequent diagnosis, comprising between 42% and 97% cases of parkinsonism [37,38,39,40,41,42,43]. The prevalence and incidence of PD vary widely due to differences in study methodologies, diagnostic criteria, case identification approaches, data collection techniques, and population demographics [37]. The dominance of PD cases limits the number of non-PD samples available for model training, reducing the statistical power and generalizability of these models. Third, the disparity in prevalence results in a highly imbalanced classification problem, where the larger PD group dominates the dataset. This imbalance often biases the ML models towards prioritizing the majority class, leading to their reduced performance in the minority class [44,45]. Finally, the heterogeneity of the diseases within the non-PD group introduces additional complexity. Non-PD parkinsonism encompasses a diverse range of syndromes with distinct underlying pathologies but overlapping clinical features, making it difficult for models to identify consistent and discriminatory patterns.

Our study uses ML to differentiate PD patients from a diverse group of participants clinically diagnosed with several non-PD parkinsonian disorders, including “atypical” neurodegenerative diseases and secondary parkinsonism. We analyze data collected from a single sensor worn on the lower back during mobility tests conducted in a hallway in a traditional clinic setting by 260 PD participants and 18 participants with etiologically diverse forms of parkinsonism (non-PD). Our objective is to highlight the potential challenges encountered when ML is applied to datasets that mimic real clinical settings and to demonstrate how these challenges can be addressed. Beyond evaluating model performance, we explored the characteristics of the participants misclassified by the model, identified the most important mobility tasks for classification, and examined whether the mobility testing protocol could be simplified to rely on a single task without compromising accuracy. Furthermore, we deployed the model in four examples of challenging scenarios commonly encountered in clinical practice, where the diagnosis was uncertain at the initial visit or the diagnosis was changed during follow-up visits.

## 2. Materials and Methods

### 2.1. Participants

Our study cohort comprised 318 individuals diagnosed with parkinsonism, all of whom were recruited from the University of Maryland Movement and Memory Disorders Center (UM-MMDC) between October 2015 and March 2020 for a study on mobility analysis in individuals with parkinsonism. Among them, 293 were diagnosed with idiopathic PD, according to the UK Parkinson’s Disease Society Brain Bank Clinical Diagnostic [46], by a movement disorder specialist at the UM-MMDC. All participants were also enrolled in the Health Outcomes Measurement (HOME) study at the UM-MMDC, a naturalistic cohort study that collects patient- and clinician-reported data on motor symptoms, activities of daily living, and quality of life during routine office visits and while patients on their regular medications. Each participant underwent a comprehensive clinical evaluation following the guidelines outlined in the HOME study protocol, which have been previously detailed [10,47,48,49]. Exclusion criteria for the mobility analysis included the inability to stand or walk without assistance (Hoehn & Yahr stage, H&Y 5), other conditions not related to parkinsonism that significantly affect gait and balance, or the failure to provide informed consent during the evaluation. Additionally, 1 PD participant was unable to complete the study procedures and was subsequently excluded, and 30 other PD participants were excluded due to missing data. All participants provided their informed consent prior to any study procedures. The study protocols for both the HOME study and the mobility analysis in the parkinsonism study were approved by the University of Maryland Institutional Review Board. All clinical neurologists involved in the evaluation and diagnosis of patients are fellowship-trained and board-certified movement disorder specialists, who use published criteria to diagnose different forms of non-PD parkinsonism (including MSA, PSP, DLB, CBS, ET, and DIP) [50,51,52,53,54,55]. Participants were enrolled during routine outpatient visits to the UM-MMDC and underwent comprehensive clinical assessments following the HOME study protocol, as detailed in our previous research [56].

### 2.2. Experimental Procedure

**Figure 1:**
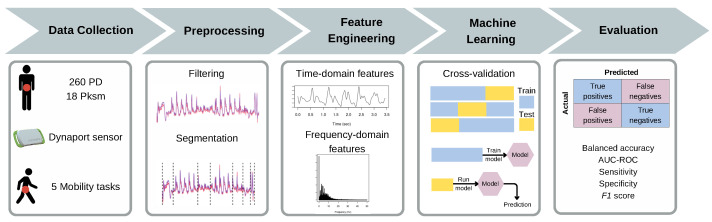
Study workflow. Pksm: non-PD parkinsonism; AUC-ROC: area under the receiver operating characteristic curve.

Using a wearable sensor, an assessment of mobility was performed following a methodology described previously [15,57,58]. Participants performed five specific mobility tasks: a 32-foot walk, standing with their eyes open for 20 s, standing with their eyes closed for 20 s, two trials of the Timed Up and Go (TUG) test, and two trials of the cognitive TUG (cogTUG). Mobility data were collected using a small (106.6 × 58 × 11.5 mm), lightweight (55 g) sensor device (Dynaport MT, McRoberts B.V., The Hague, The Netherlands, technical specifications: https://www.mcroberts.nl/products/movetest/, accessed on 7 January 2025) attached to their lower back using a waist belt. This device, equipped with a triaxial accelerometer (range: ±8 g; resolution: 1 mg) and a triaxial gyroscope (range: ±2000 dps; resolution: 70 dps), measured acceleration and rotation along three axes (vertical, mediolateral, anteroposterior) at a rate of 100 Hz. The sensor was remotely controlled via Bluetooth, and digital markers were placed at task start and end points to identify each mobility task later on. After each test, data were transferred to a secure server and analyzed using a custom-made Matlab (version 9.1) GUI. The accelerometry patterns and digital markers were used to segment the data for further analysis. The details of the mobility assessment methodology, devices used, and data acquisition and retrieval have been described in our previous work [56]. Figure 1 presents an overview of the study’s workflow.

### 2.3. Feature Engineering

To reduce noise in the sensor signals, we implemented a low-pass, 20 Hz, zero-lag, 4th order Butterworth filter [59]. The order and cutoff frequency of the filter were selected based on common practices in the literature [60,61,62,63] and further refined through exploratory data analysis. We then segmented the complex motor tasks, such as TUG, cogTUG, and the 32-foot walk, into their respective subtasks. We extended a previous method [64] to identify turns, sit-to-stand, stand-to-sit, and walking segments using the trapezoidal integration [65] of sensor data. Stationary periods were removed to correct timing discrepancies. For a detailed description of the segmentation approach, we refer readers to our previous work [56].

For every participant and task, we computed various sets of features across the six recorded channels, including statistical measures consisting of 22 frequency-domain features (Supplementary Table S1), 42 time-domain features (Supplementary Table S2), the top 10 power amplitudes from the Discrete Fourier Transform (DFT) and Lomb-Scargle Periodogram (LSP) [66,67], and 3 time-domain features computed across the signals from each pair of the six channels (Supplementary Table S3).

Each participant was characterized by a total of 32,514 sensor-derived features computed from the six channels of the 32-foot walk segmented subtasks, standing with closed eyes task, standing with open eyes task, TUG segmented subtasks (from the first trial, the second trial, and the mean and difference between corresponding features from trials), and cogTUG segmented subtasks (from the first trial, the second trial, and the mean and difference between corresponding features from both trials). Before executing the ML models, preprocessing procedures were applied to the data, including the elimination of variables with constant or infinite values and the imputation of missing values using the median of the observed values.

### 2.4. Mutual Information-Based Feature Selection for EasyEnsemble (MIEE)

We used the mutual information-based feature selection for EasyEnsemble (MIEE) [68] algorithm due to its strong performance on highly imbalanced datasets, such as ours (260 PD and 18 non-PD parkinsonism participants). This method combines EasyEnsemble [69] and feature selection to effectively address class imbalances. EasyEnsemble creates multiple independent balanced subsets by undersampling the majority class, trains classifiers on these subsets, and combines their predictions using ensemble methods. To improve accuracy, MIEE incorporates feature selection by calculating the mutual information (MI) [70] scores between features and the target class, ranking features based on their scores, and selecting the most informative ones. This process helps reduce dimensionality and mitigates the risk of model overfitting. The process begins by generating balanced subsets, applying MI-based feature selection to each subset, training classifiers on these subsets using selected features, and aggregating the predictions of the generated classifiers through ensemble techniques. In our implementation, we created five balanced subsets, identified the top 30 features for each subset using MI scores, and trained a Random Forest (RF) model [71] with 1000 trees for each subset using the R package randomForest. The final predictions for each participant were obtained by averaging the prediction probabilities of the five classifiers.

### 2.5. Machine Learning Model

We used a three-fold cross-validation technique to create training and test splits for training our our models using stratified sampling. This process was repeated five times with different random seeds to improve robustness. Cross-validation ensured that each data point appeared exactly once in the test set, which was necessary given the small sample size of non-PD participants, making a separate test set impractical. Repeating this procedure with different random seeds increases the diversity of the train–test splits and reduces the likelihood of overfitting. The final class for each participant was determined based on the class most commonly predicted by the five replicates. Within each fold, the model described in Section 2.4 was trained on the training set and tested on the corresponding test set to prevent data leakage and ensure generalization [72].

Several steps were taken to address the imbalance between the PD and non-PD parkinsonism groups. First, we used stratified sampling when creating training and testing sets to maintain a uniform class representation in the cross-validation folds. Second, the MIEE algorithm was used due to its demonstrated effectiveness in handling highly imbalanced datasets [68]. Third, the performance of the models was evaluated using balanced accuracy, sensitivity, specificity, F_1_ score, and AUC-ROC metrics. These metrics are considered more appropriate for evaluating models in cases of imbalanced datasets compared to overall accuracy, which can be misleading due to its tendency to prioritize common classes over rare ones [73].

### 2.6. Group Feature Importance

We employed the notion of group feature importance to evaluate the predictive contribution of various mobility tasks. Group feature importance involves assessing the combined information of a set of features within a specific category. All features derived from individual mobility tasks were aggregated into five distinct groups, each corresponding to one of the five mobility tasks. To assess the importance of each group, we adapted the permutation importance method for feature groups within the framework of random forests, based on a previously outlined approach [74]. For each feature group, the grouped variables were simultaneously permuted, and the resulting mean decrease in accuracy before and after permutation was calculated.

### 2.7. Statistical Analysis

The data analyses were conducted using R version 4.2.3 (15 March 2023) and RStudio version 2023.6.0.421. We used a two-tailed Student’s t-test to compare continuous variables and Pearson’s $\chi^{2}$ test to compare categorical variables between the two groups. Continuous variables were presented as a mean ± standard deviation (SD), and actual *p*-values for the statistical tests were reported. For each of the models’ performance metrics, we presented both the calculated values and their corresponding 95% two-sided confidence intervals (CIs). These intervals were obtained through balanced bootstrap resampling using 10 K replicates and were adjusted using bias correction and acceleration to enhance our control over bias and skewness in the bootstrap distribution. We used the boot function in the R package boot version 1.3–30 [75,76] to generate the bootstrap replicates and the boot.ci function from the same package to generate the CIs.

## 3. Results

Wearable sensor data were collected from a cohort of 260 individuals diagnosed with PD (87% were receiving antiparkinsonian medication) and 18 participants diagnosed with other forms of parkinsonism. In the latter group, there were four cases of parkinsonian-type MSA (MSA-P), three cases of PSP, three cases of DLB, two cases of CBS, two cases of ET, and one case of DIP. Three patients with unspecified parkinsonism were also included. Table 1 provides the characteristics of the study cohort.

Each participant performed five motor tasks, including a 32-foot walk, standing with eyes open, standing with eyes closed, two trials of the TUG, and two trials of the cogTUG (see Section 2.2 for details). For simple tasks such as standing with eyes open and standing with eyes closed, we computed a set of frequency-domain and time-domain features. For more complex tasks such as the 32-foot walk, TUG, and cogTUG, we initially segmented these into their respective subtask components, from which features were then derived for each subtask signal (see Section 2.3 for details). Then, we applied the ML model described in Section 2.5, using features from all five tasks, to predict the final class for each participant. A summary of the process is provided in Figure 1.

### 3.1. Feature Importance Analysis

Across the replicates, folds, and balanced subsets of the model, 82.4% of the selected features were derived from the TUG task, including the first trial, second trial, mean, and difference between corresponding features from both trials. Among these TUG-derived features, 42.5% were calculated specifically from the sit-to-stand phase, while the remaining features were equally distributed across the turn, stand-to-sit, and walking phases. These features captured acceleration and rotational metrics along the vertical, mediolateral, and anteroposterior axes. Additionally, 11% of the selected features were calculated from the cogTUG task, while the remaining features were derived from the 32-foot walk, stand with eyes open, and stand with eyes closed tasks.

**Figure 2:**
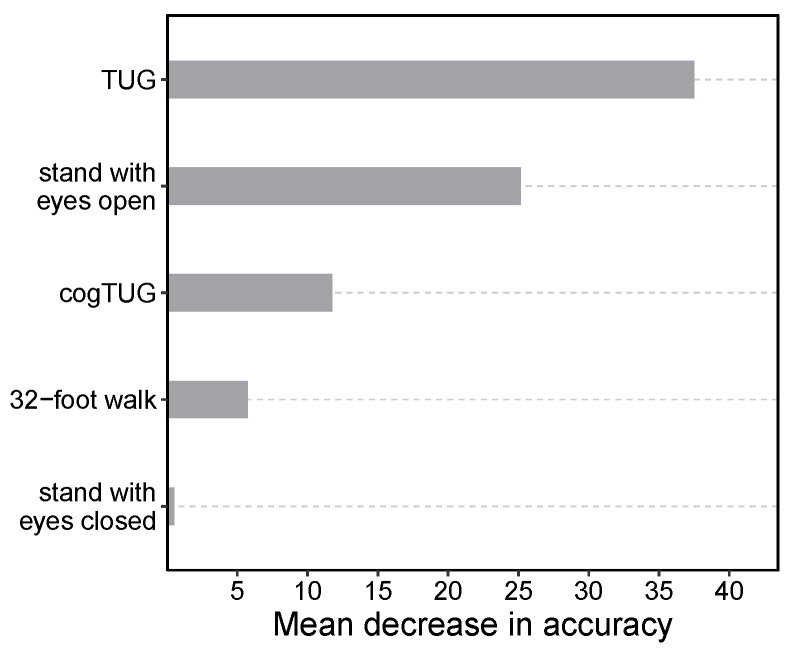
Group feature importance for the classification of Parkinson’s disease and non-PD parkinsonism. The ranking of groups is determined based on their importance, which is computed by simultaneously permuting features within each group and measuring the average decrease in accuracy between the original and permuted data. Under the null hypothesis, which posits no association between the group of predictor variables and the model’s prediction, permutation should result in no impact on predictive performance.

The value of the information from the TUG task was also demonstrated by conducting a group feature importance analysis that included the five mobility tasks. The features derived from the TUG task emerged as the most influential for the classifier, surpassing the importance of other feature groups by a margin of at least 43% (Figure 2).

### 3.2. Model Performance

The trained model achieved a balanced accuracy (average accuracy of both classes) of 72.9% (confidence interval [CI] 60.2%, 81.4%), with an area under the receiver operating characteristic curve (AUC-ROC) of 0.73 (CI 0.63, 0.83). Its sensitivity, specificity, and F_1_ score were 0.68 (CI 0.62, 0.74), 0.78 (CI 0.50, 0.93), and 0.80 (CI 0.76, 0.84), respectively (Table 2, first column). Table 3a shows the confusion matrix of the classifier.

To streamline the complexity of our models, we sought to construct classifiers based solely on features derived from the most informative mobility task. To that end, we focused exclusively on TUG features, given its high ranking in the feature importance analysis. We then implemented the ML model detailed in Section 2.5 using these TUG-specific features. The TUG-only model outperformed the initial model, which included all mobility tasks, achieving a 5.3% improvement in balanced accuracy (Table 2). Its corresponding confusion matrix is presented in Table 3b. This finding suggests that sensor data from the TUG mobility task may be sufficient to achieve a satisfactory performance in differentiating PD from other forms of parkinsonism. Consequently, our subsequent analysis focused on the TUG-only model, given its simplicity and good performance in terms of balanced accuracy compared to the all-tasks model. The features selected for the TUG-only model were distributed across the components of the TUG task as follows: 36.3% from the sit-to-stand phase, 24.7% from the stand-to-sit phase, 23.7% from the turn phase, and 15.3% from the walking phase.

### 3.3. Misclassification Analysis

To gain insight into the characteristics of participants misclassified by the TUG-only model, we conducted comparisons of various demographic and clinical scores between correctly classified and misclassified participants with PD or non-PD parkinsonism. Two-tailed Student’s t-tests were used to compare continuous variables, and Pearson’s $\chi^{2}$ tests were used to compare categorical variables across the groups. The summarized comparison results are presented in Table 4. The 70 PD participants falsely classified as having non-PD parkinsonism exhibited significantly (*p* < 0.05) higher Unified Parkinson’s Disease Rating Scale [77] part III (UPDRS_PIII) motor scores, lower Montreal Cognitive Assessment (MoCA) [78] scores, higher Cumulative Illness Rating Scale-Geriatrics (CIRS-G) [79] scores, greater age, and longer durations of the disease compared to their 190 correctly classified counterparts. Further examination of the distribution of misclassified PD participants across different H&Y [80,81] stages revealed a higher-than-expected number of misclassifications at more severe stages (H&Y = 3 and 4). Additionally, the three misclassified non-PD participants had significantly lower UPDRS_PIII motor scores than the 15 correctly classified non-PD participants. These results indicate that the models tend to misclassify PD participants with more severe motor symptoms and non-PD participants with milder motor symptoms. No significant differences in MoCA scores, CIRS-G scores, age, disease duration, or sex were observed between the correctly and incorrectly classified non-PD participants.

Given the heterogeneous nature of the disorders within the non-PD parkinsonism group, we sought to determine whether there is a specific type of parkinsonism that is more similar to PD and, therefore, more likely to be misclassified by the model. To investigate this, we examined the types of parkinsonism in the three misclassified non-PD participants. We found that one participant had DLB, one had ET, and one had unspecified parkinsonism. This suggests that no particular type of non-PD parkinsonism is more likely to be misclassified as PD. However, the small number of misclassified participants precludes use reaching a definitive conclusion in this regard. Similarly, to explore whether misclassified PD participants share similarities with specific types of non-PD parkinsonism, we used the RF proximity matrix to determine the type of non-PD parkinsonism most similar to each misclassified PD participant (see the Supplementary Results for details). We found that the misclassified PD participants were relatively evenly distributed across the different types of non-PD parkinsonism (Supplementary Table S4), indicating that they did not show a particular similarity to one type of parkinsonism over others.

### 3.4. Model Predictions for Challenging Cases

To evaluate the performance of the model in particularly challenging scenarios, we analyzed its predictions for four cases initially assessed as a parkinsonian syndrome (no specific diagnosis) at the time of initial gait testing. Three of these participants (between 54 and 69 years old at study enrollment) presented with balance problems early in their disease course, prominent dysautonomia (including urinary urgency and orthostatic hypotension), and mostly symmetrical parkinsonism without a clear response to levodopa. In all three cases, the treating movement disorder neurologists concluded that PD and MSA were equally likely at that point. Therefore, patients were classified as having a parkinsonian syndrome. Over the next one to four years, all three patients showed a robust response to levodopa, slow disease progression, and dysautonomia that was less prominent at follow-up. Therefore, the diagnosis was changed to idiopathic PD for each of these three participants. Due to their indeterminate diagnostic labels (MSA vs. PD) at the time of enrollment, all three participants were excluded from the training dataset and were instead classified by the models trained on the non-excluded participants. We used the TUG-only model to determine the mean prediction score for the three participants. One participant (participant a, H&Y = 2) was classified as having PD by the model, with a score in the 18.6 percentile of all PD-classified participants. The other two participants were classified as having non-PD parkinsonism by the model. One of them (participant b, H&Y = 3) was assigned to the non-PD group with a model score in the 35.2 percentile of all participants classified as having non-PD parkinsonism. The other participant (participant c, H&Y = 2) was also classified as having non-PD parkinsonism, but the model score was only in the 12.1 percentile among the participants classified as having non-PD parkinsonism.

In the fourth challenging case, with a change in diagnosis over time, the participant was a 68-year-old patient (participant d, H&Y = 2), with prominent hypophonia, stuttering, and symmetrical parkinsonism without a clear response to levodopa. In the absence of clear features suggestive of a specific form of parkinsonism, the treating neurologist diagnosed that person with an unspecified parkinsonian syndrome. Over the next four years, the patient reported significant worsening of his motor symptoms after stopping levodopa and developed typical levodopa-induced motor fluctuations and choreiform dyskinesias after re-starting levodopa. At that point, the movement disorder specialist made the diagnosis of idiopathic PD. Since this participant was diagnosed with an unspecified parkinsonian syndrome at the time of enrollment and mobility testing, they were included in our models as a non-PD parkinsonism participant. However, both the all-tasks and TUG-only models misclassified this participant as having PD, agreeing with the diagnosis made by the movement disorder specialist during follow-up evaluations.

### 3.5. The Performance of Alternative Models

In addition, we evaluated the performance of various ML models and sampling techniques using TUG-only features. Each of them was tested within the three-fold cross-validation and five-repeat framework: features were selected from the training set, the model was trained on the training data, and its performance was evaluated on the corresponding test set using the same features selected from the training data. First, we applied an unsupervised RF to rank the features by importance, selected a subset of the top 30 features that maximized the balanced accuracy of the training set, and trained a final RF model using the selected features (Table S5a; Table 5, first column). Second, we calculated the mutual information between all features and the target variable, ranked them by mutual information scores, and selected the top 30 features to train an RF model (Table S5b; Table 5, second column). Third, we implemented the feature selection method described in a previous work [82], which calculates the F_1_ scores for the features using a decision tree classifier. The features were ranked according to their scores, and the top 30 features were used to train an RF model (Table S5c; Table 5, third column). Fourth, we employed a supervised RF approach to rank features by importance, selecting a subset that minimized the Akaike information criterion (AIC) [83] for the training set. The final RF model was built using the selected features (Table S5d; Table 5, fourth column).

To address the class imbalance between PD and non-PD parkinsonism, we explored the impact of sampling the training set by undersampling the PD class and oversampling the non-PD class, as proposed in previous research [84]. We then employed three sampling placement strategies described in another work [85]. For each approach, the features were ranked using RF importance scores, and a subset was selected that minimized the AIC of the model. The approaches were as follows: 1. sample the training set first, followed by feature selection and RF model training using the original training data (Table S5e; Table 6, first column); 2. sample the training set first, performing feature selection, and train the RF model on the sampled data (Table S5f; Table 6, second column); and 3. perform feature selection on the original training set, followed by sampling and training of the RF model on the sampled data (Table S5g; Table 6, third column).

Additionally, we implemented random undersampling of the PD group to match the size of the non-PD parkinsonism group each time an RF model was constructed. However, none of these models surpassed the performance of our suggested model (Section 2.5).

## 4. Discussion

We demonstrate the potential of the machine learning analysis of sensor data collected during mobility testing from a single wearable accelerometer and gyroscope to distinguish PD from other forms of parkinsonism. The inherent similarities between these movement disorders make distinguishing idiopathic PD from other parkinsonian conditions a well-recognized diagnostic challenge for clinicians [1]. Based on findings from a previous meta-analysis, the balanced accuracy of the clinical diagnosis of PD was reported to be 69.5% when conducted primarily by non-experts, 78.4% when conducted by movement disorders experts during their initial assessment, and 82.4% for their diagnosis after follow-up [8]. Despite the overlap in motor symptoms between the groups, our TUG-only model achieved a balanced accuracy of 78.2%, approaching the diagnostic accuracy of experts during their initial assessments. Expert clinicians reported a sensitivity of 81.8% and specificity of 74.9% for their initial diagnoses [8], while our TUG-only model achieved a sensitivity of 73.1% and specificity of 83.3%. This suggests that such a model could serve as an additional diagnostic “expert”, which is particularly valuable for challenging cases, by providing an objective assessment based on a single mobility task and without requiring the full clinical evaluation used by clinicians. Additionally, wearable sensors offer additional benefits for remote monitoring and early-stage diagnosis, presenting a data-driven alternative to traditional clinical assessments [15].

One substantial challenge limiting model performance is the small sample size of the non-PD group. This imbalance is a common issue in clinical settings, given the higher prevalence of PD compared to other parkinsonian disorders [36,37]. To address this, we applied a sampling approach recommended in a prior study [84], which combined the undersampling of the majority (PD) class, using the Neighborhood Cleaning Rule (NCL) [86], and the oversampling of the minority (non-PD) class using the synthetic minority oversampling technique (SMOTE) [87]. We integrated these sampling techniques with three strategies proposed in another study, comparing model performance when sampling occurred before or after feature selection and when classifiers were built using original (unsampled) or sampled data [85]. However, none of these approaches improved upon the performance of our suggested model (Supplementary Table S5e–g).

Other challenges included significant class imbalance and heterogeneity within the non-PD group. The steps taken to address this class imbalance, described in Section 2.5, included stratified sampling for the training and testing sets in cross-validation folds, using the MIEE algorithm for its effectiveness in imbalanced datasets [68] and evaluating models with appropriate metrics for imbalanced data, such as balanced accuracy [73]. Although the heterogeneity of the non-PD group negatively affected model performance, it can also be seen as a strength of this study, because it represents an actual clinical population with a mixed group of parkinsonism patients, where all different forms of non-PD parkinsonism must be considered as differential diagnoses. Studies that only compare PD with one form of non-PD parkinsonism (e.g., PSP [88]) have a clear advantage in terms of statistics and the homogeneity of their two groups, but have the disadvantage of analyzing a somewhat artificially simplified situation. The same principles apply to the heterogeneity within the PD group, as our dataset includes a large PD cohort with varying stages of the disease, further reflecting the diversity encountered in clinical practice.

Analysis of group and individual feature importance identified the TUG task as the most important mobility task for distinguishing PD participants from those with other forms of parkinsonism (Figure 2). The model using only TUG task features achieved a 5.3% higher balanced accuracy compared to the model incorporating features from all five mobility tasks. Previous studies have emphasized the significance of gait and sway tasks [16,19,89,90]. However, in the context of our study and the included mobility tasks, we found that gait and sway tasks were less important compared to the TUG. The TUG encompasses a greater diversity of movements, including movements that appear to be especially sensitive to parkinsonism, such as rising from a chair, sitting down, and turning $180^{\circ }$. This observation is reflected in the features of the TUG selected by our model, which were primarily derived from the sit-to-stand, stand-to-sit, and turn components. Those specific parts of the TUG might be particularly sensitive to atypical forms of parkinsonism, which tend to affect axial motor function earlier and more severely than idiopathic PD [18]. Although sensor-derived features from the TUG task identified it as the most informative, statistical tests (Student’s *t*-test) did not reveal significant differences between the UPDRS_III axial items (e.g., 3.9, 3.10, or 3.12) in the PD and non-PD groups.

A study highlighting TUG as one of the mobility tests with superior discriminative abilities between PD and atypical parkinsonism, with an AUC of 0.77 [18], aligns well with the results from our TUG-only model (AUC = 0.78). Our results show a better performance compared to a previous study that reported a balanced accuracy of 72.42% for distinguishing PD from a diverse group of non-PD parkinsonism by combining smartwatch data from movement-based assessments with an electronic questionnaire [24]. When questionnaire data were excluded, as in our models, the balanced accuracy in that study decreased to 69.18%.

Using a single sensor and a single task (TUG in our study) significantly simplifies both the mobility testing process in clinical settings and the data processing and analysis required for the model. This streamlined approach enhances the feasibility of adopting sensor-based mobility testing as a diagnostic tool in routine evaluations of movement disorders. These findings complement our previous work, which demonstrated that the clinical protocol for distinguishing PD from a control group could be simplified using a single sensor and task [56], with further simplification achieved by reducing the number of trials per task to just one [91].

Our analysis of the participants misclassified by the TUG model revealed a tendency to misclassify PD participants with more severe disease symptoms and non-PD participants with milder symptoms (Table 4). Although the non-PD group in this study had a shorter disease duration than the PD group, they displayed more severe motor symptoms, as reflected by their higher UPDRS_PIII scores (Table 1). Matching both groups by disease duration would result in differing severity levels, as other forms of parkinsonism are known to progress more rapidly than PD [92,93].

For a small group of study participants, the diagnosis changed from the time of the initial mobility testing to their clinical follow-up visit(s). We explored the characteristics of those participants more closely. Among them, three participants (excluded from the models) initially had indeterminate diagnostic labels (MSA vs. PD) but were later diagnosed with idiopathic PD during follow-up visits. Of these, our TUG-only model correctly classified one participant as having PD, while the other two were predicted to have non-PD parkinsonism, although their model scores were relatively low among those classified in the non-PD group. Another participant, initially diagnosed with an unspecified parkinsonian syndrome, was later confirmed to have idiopathic PD at follow-up. This participant, included in our models as a non-PD case, was misclassified as having PD by the model, aligning with the revised clinical diagnosis made at follow-up. These cases serve as examples of challenging scenarios encountered in real clinical settings, where diagnostic decisions can be as difficult for ML models as they are for clinicians.

In that context, it is also important to remember that there is no true diagnostic “gold standard” for these conditions, apart from a postmortem pathological examination of brain tissue. It is therefore quite possible that in a cohort of close to 300 participants across the two groups, a few participants may have been clinically misdiagnosed and, in fact, categorized correctly by our ML approach. This is an inherent problem for this type of analysis that will only be overcome once reliable and specific diagnostic biomarkers for the different forms of parkinsonism have been established.

A limitation of our work is the small sample size of our non-PD parkinsonism group, which may limit the generalizability of our findings. Subsequent research efforts should prioritize validating our results within larger cohorts to enhance the robustness of our conclusions. Additionally, the small sample size prevented us from addressing the heterogeneity issue by developing a multiclass classifier, where each form of parkinsonism could be represented by a separate class. Furthermore, given the differences in UPDRS_PIII, H&Y scores, age, and other clinical measures between the correctly classified and misclassified participants, stratifying the model by one or more of these characteristics could potentially improve its performance. However, due to the limited sample size, this approach was not feasible in our study. Future studies should also explore the impact of including clinical measures (e.g., UPDRS, H&Y, disease duration) and other key diagnostic factors (e.g., severity of dysautonomia, early cognitive impairment, early falls, and vertical eye movement abnormalities) in ML models to determine whether incorporating these features improves their diagnostic accuracy.

## 5. Conclusions

Our study demonstrates the potential of machine learning applied to wearable sensor data to distinguish PD from other parkinsonian disorders while using a single sensor. The TUG task was the most valuable mobility task for our classifier, and using only this task would simplify mobility testing protocols. The TUG-only model achieved a balanced accuracy comparable to that of expert clinicians. We also discussed the challenges encountered when applying ML to datasets that reflect real clinical settings and presented strategies to address these issues.

## Figures and Tables

**Table 1:** Characteristics of the study cohort. UPDRS: Unified Parkinson’s Disease Rating Scale. H&Y: modified Hoehn and Yahr.

Feature	PD (n=260)	non-PD Parkinsonism (n=18)	*p*
Age (years, $\bar{x}\pm SD$)	66.8 ± 9.3	68.7 ± 8.2	0.377
Gender (%male)	62.3	38.9	0.093
Height (cm, $\bar{x}\pm SD$)	172.2 ± 10.4	165.3 ± 13.8	0.059
UPDRS (total, $\bar{x}\pm SD$)	35.1 ± 17.1	47.9 ± 22.4	0.033
UPDRS (motor-part III, $\bar{x}\pm SD$)	22.1 ± 11.8	28.8 ± 14.9	0.085
Disease duration (years, $\bar{x}\pm SD$)	7.8 ± 6.5	1.7 ± 2.1	4.2 × 10^−10^
H&Y ($\bar{x}\pm SD$)	2.2 ± 0.62	2.9 ± 0.77	0.005
stage 1 (n)	12	0	
stage 1.5 (n)	4	0	
stage 2 (n)	168	6	
stage 2.5 (n)	35	3	
stage 3 (n)	24	5	
stage 4 (n)	17	4	

**Table 2:** Classification results of all-tasks and TUG-only models using participants with Parkinson’s disease (PD) and non-PD parkinsonism. CI: 95% confidence interval.

	All Tasks	TUG-Only
Balanced accuracy (CI) [%]	72.9 (60.2, 81.4)	78.2 (65.7, 85.6)
AUC-ROC (CI)	0.73 (0.63, 0.83)	0.78 (0.69, 0.87)
Sensitivity (CI)	0.68 (0.62, 0.74)	0.73 (0.67, 0.78)
Specificity (CI)	0.78 (0.50, 0.93)	0.83 (0.56, 0.95)
F_1_ score (CI)	0.80 (0.76, 0.84)	0.84 (0.80, 0.87)

**Table 3:** Confusion matrices of the classifiers using participants with Parkinson’s disease (PD) and non-PD parkinsonism (Pksm). Rows represent actual classes and columns represent predictions.

(a) All Tasks			(b) TUG-Only		
	PD	Pksm		PD	Pksm
PD	177	83	PD	190	70
Pksm	4 *	14	Pksm	3 *	15

* One of the misclassified Pksm participants was later diagnosed with PD.

**Table 4:** Clinical characteristics of participants with Parkinson’s disease (PD) and non-PD parkinsonism correctly and incorrectly classified by the TUG-only model.

	PD			Non-PD Parkinsonism		
	**Correctly Classified**	**Incorrectly Classified**	p	**Correctly Classified**	**Incorrectly Classified**	p
UPDRS_PIII	19.7 ± 10.9	28.7 ± 11.4	3.8 × 10^−8^	30.5 ± 14.9	17.7 ± 5.7	0.016
MoCA	27.6 ± 2.8	26.5 ± 3.5	0.02	26.3 ± 4.9	23.3 ± 2.1	0.127
CIRS-G	4.5 ± 3.2	5.7 ± 3.9	0.035	8.0 ± 4.6	7.2 ± 3.6	0.73
Age	66.0 ± 8.9	69.1 ± 9.9	0.023	67.6 ± 8.1	74.0 ± 5.2	0.15
Disease duration	7.1 ± 5.9	8.7 ± 6.9	0.038	2.5 ± 2.3	2.3 ± 2.5	0.47
Sex (% male)	59.5	70.0	0.184	33.3	100.0	0.053
H&Y (n)			1.3 × 10^−6^			0.40
1	10	2		0	0	
1.5	3	1		0	0	
2	138	30		4	2	
2.5	27	8		2	1	
3	8	16		5	0	
4	4	13		4	0	

**Table 5:** Classification results of alternative machine learning models using TUG-only features. RF: random forest; FS: feature selection; bacc: balanced accuracy; MI: mutual information; DT: decision tree; AIC: Akaike information criterion.

	Unsupervised RF FS with Max Bacc	MI-Based Ranking for Top FS	F_1_ Ranking Using DT for Feature Scoring	Supervised RF FS with Min AIC
Balanced accuracy (%)	67.8	68.2	68.3	52.1
AUC-ROC	0.68	0.68	0.68	0.52
Sensitivity	0.80	0.92	0.87	0.93
Specificity	0.56	0.44	0.50	0.11
F_1_ score	0.87	0.94	0.91	0.93

**Table 6:** Classification results of sampling techniques using TUG-only features. FS: feature selection.

	Sampling Before FS, Model Trained on Original Data	Sampling Before FS, Model Trained on Sampled Data	FS Before Sampling, Model Trained on Sampled Data
Balanced accuracy (%)	56.4	49.6	59.4
AUC-ROC	0.56	0.50	0.59
Sensitivity	0.96	0.99	0.97
Specificity	0.17	0.00	0.22
F_1_ score	0.95	0.96	0.96

## Data Availability

De-identified demographic, clinical, and sensor-derived data have been archived in the Digital Repository at the University of Maryland (DRUM), https://doi.org/10.13016/2qhy-961s. The code used in this research paper is also accessible via the same link. The provided notebook contains the code necessary to execute each aspect of the signal processing and machine learning models. Comprehensive instructions for configuring the required environment and executing the code are outlined in the README file.

## Supplementary Materials

The following supporting information can be downloaded at: https://www.mdpi.com/article/10.3390/biomedicines13030572/s1, Table S1: Frequency domain features. The power spectrum is obtained using the Discrete Fourier Transform (DFT) of the signal with a fast algorithm, the Fast Fourier Transform (FFT). Table S2: Time domain features. Table S3: Cross-time domain features. Table S4: The count of misclassified participants with Parkinson’s disease (PD) that closely resemble each type of non-PD parkinsonism. Table S5: Confusion matrices of the classifiers using Parkinson’s disease (PD) and non-PD parkinsonism (Pksm) participants using features from TUG mobility task. Rows represent actual class and columns represent predictions. References [94,95,96,97,98,99,100,101,102,103,104,105,106,107,108,109,110,111,112,113,114,115] are cited in the Supplementary Materials.
