# Supplementary material for: Applying Wearable Sensors and Machine Learning to the Diagnostic Challenge of Distinguishing Parkinson’s Disease from Other Forms of Parkinsonism
Source: Biomedicines. 2025 Feb 25;13(3):572. doi: 10.3390/biomedicines13030572 (PMC11940150; doi:10.3390/biomedicines13030572)
Supplement: Supplementary file 1 [file biomedicines-13-00572-s001.zip › biomedicines-3452981-supplementary.pdf]

Supplementary material for applying wearable sensors and machine learning to the diagnostic challenge of distinguishing Parkinson's disease from other forms of parkinsonism

Rana M. Khalil[1], Lisa M. Shulman[2], Ann L. Gruber-Baldini[3], Stephen G. Reich[2], Joseph M. Savitt[2], Jeffrey M. Hausdorff[4,5,6,7,8], Rainer von Coelln[2,*], and Michael P. Cummings[1,*]

[1]Center for Bioinformatics and Computational Biology, University of Maryland, College Park, Maryland, USA
[2]Department of Neurology, University of Maryland School of Medicine, Baltimore, Maryland, USA
[3]Department of Epidemiology and Public Health, University of Maryland School of Medicine, Baltimore, Maryland, USA
[4]Center for the Study of Movement, Cognition, and Mobility, Neurological Institute, Tel Aviv Medical Center, Tel-Aviv, Israel
[5]Department of Physical Therapy, Sackler Faculty of Medicine, Tel Aviv University, Tel Aviv, Israel
[6]Sagol School of Neuroscience, Tel Aviv University, Tel Aviv, Israel
[7]Rush Alzheimer's Disease Center, Rush University Medical Center, Chicago, IL, USA
[8]Department of Orthopedic Surgery, Rush University Medical Center, Chicago, IL, USA
[*]Correspondence: rvoncoelln@som.umaryland.edu (R.vC), mcummin1@umd.edu (M.P.C)

Contents

Supplementary Results **9**

Supplementary Files **9**

Supplementary Tables

S1 Table S1 2
S2 Table S2 4
S3 Table S3 7
S4 Table S4 8
S5 Table S5 8

Table S1. Frequency domain features. The power spectrum is obtained using the Discrete Fourier Transform (DFT) of the signal with a fast algorithm, the Fast Fourier Transform (FFT) [1]. Variables: f_j is the frequency of the spectrum at frequency bin j; P_j is the power spectrum at frequency bin j; and M is the length of the frequency bin.

Name	Description	Mathematical equation	References
MNF	Mean frequency	$\sum_{j=1}^{M} f_j P_j \ / \ \sum_{j=1}^{M} P_j$	[2, 3, 4]
MDF	Median frequency	$\frac{1}{2} \sum_{j=1}^{M} P_j$	[2, 3, 4]
PSD	Power spectrum deformation	$\frac{\sqrt{\frac{M_2}{M_0}}}{\frac{M_1}{M_0}}$; $M_n = \sum_{j=1}^{M} f_j P_j$	[3, 5]
FI	Freeze index	$\sum_{j=3}^{8} P_j \ / \ \sum_{j=0.5}^{3} P_j$	[6, 4]
ENT	Entropy	$-\sum_{j=1}^{M} Pr_j log(Pr_j)$; $Pr_j = \frac{P_j}{\sum_{j=1}^{M} P_j}$	–
TTP	Total power	$\sum_{j=1}^{M} P_j$	[2, 4]
MNP	Mean power	$\sum_{j=1}^{M} P_j \ / \ M$	[2, 4]
PKF	Peak frequency	$f_{argmax(P_j)\ ,\ j=1...M}$	[2, 4]
PKF	Peak frequency	$max(P_j)$, $j = 1...M$	[2]
FR	Frequency ratio	$\frac{max(f_j)}{min(f_j)}$, $j = 1...M$	[2, 4]
PSR	Power spectrum ratio	$\frac{P_0}{P} = \sum_{f_0-n}^{f_0+n} P_j \ / \ \sum_{j=-\infty}^{\infty} P_j$<br>$f_0$: value of PKF, n: integral limit.	[2]
SM	Spectral moments	$SM_i = \sum_{j=1}^{M} f_j^i P_j$, $i = 1, 2, 3$	[2, 5, 4]
VR	Variance	$\frac{1}{M-1} \sum_{j=1}^{M} (P_j - MNP)^2$	–
SD	Standard deviation	$\sqrt{\frac{1}{M-1} \sum_{j=1}^{M} (P_j - MNP)^2}$	–
SS	Skewness	$\frac{1}{M-1} \sum_{j=1}^{M} (P_j - MNP)^3 \ / \ SD^3$	–
SK	Kurtosis	$\frac{1}{M-1} \sum_{j=1}^{M} (P_j - MNP)^4 \ / \ SD^4$	–
SBW	Spectral bandwidth	$\frac{\sum_{j=1}^{M} (f_j - MNF)^2 P_j}{TTP}$	–
SR	Spectral roll-off	$c \sum_{j=1}^{M} P_j$	–
VCF	Variance of central frequency	$\frac{SM_2}{SM_0} - \left(\frac{SM_1}{SM_0}\right)^2$	[2]

MSE	Mean spectral energy	$\sum_{j=1}^{M} \lvert P_j \rvert^2 \,/\, M$	[7, 8]
DPR	Maximum to minimum drop in power density	$\dfrac{\text{highest mean power density value}}{\text{lowest mean power density value}}$	[5, 3]
SN	Signal to noise ratio	$\dfrac{\sum P_j \text{ in the upper 20\% frequency range}}{TTP}$	[5, 3]

Table S2. Time domain features. Variables: x_n represents n^{th} sample of the signal; and N is the length of the signal.

Name	Description	Mathematical equation	References
MN	Mean	$\frac{1}{N}\sum_{n=1}^{N} x_n$	[9, 3, 10]
VR	Variance	$\frac{1}{N-1}\sum_{n=1}^{N}(x_n - MN)^2$	[3]
SD	Standard deviation	$\sqrt{\frac{1}{N-1}\sum_{n=1}^{N}(x_n - MN)^2}$	[9, 3, 10]
SS	Skewness	$\frac{1}{N-1}\sum_{n=1}^{N}(x_n - MN)^3 \;/\; SD^3$	[9, 3, 10]
SK	Kurtosis	$\frac{1}{N-1}\sum_{n=1}^{N}(x_n - MN)^4 \;/\; SD^4$	[9, 3, 10]
IAV	Integrated absolute value	$\sum_{n=1}^{N} \lvert x_n \rvert$	[2, 4]
MAV	Mean absolute value	$\frac{1}{N}\sum_{n=1}^{N} \lvert x_n \rvert$	[2, 4]
SSI	Simple square interval	$\sum_{n=1}^{N} \lvert x_n \rvert^2$	[2, 4]
RMS	Root mean square	$\sqrt{\frac{1}{N}\sum_{n=1}^{N} x_n^2}$	[2, 4]
V3	V-order 3	$\sqrt[3]{\frac{1}{N}\sum_{n=1}^{N} \lvert x_n \rvert^3}$	[2, 4]
WL	Waveform length	$\sum_{n=1}^{N-1} \lvert x_{n+1} - x_n \rvert$	[2, 3, 4]
AAC	Average amplitude change	$\frac{1}{N}\sum_{n=1}^{N-1} \lvert x_{n+1} - x_n \rvert$	[2, 4]
DASDV	Difference absolute standard deviation value	$\sqrt{\frac{1}{N-1}\sum_{n=1}^{N-1}(x_{n+1} - x_n)^2}$	[2, 4]
MFL	Maximum fractal length	$log_{10}\left(\sqrt{\sum_{n=1}^{N-1}(x_{n+1} - x_n)^2}\right)$	[4, 11]

ZC	Zero crossing	$\sum_{n=1}^{N-1} sgn(x_n * x_{n+1}) \cap \lvert x_n - x_{n+1} \rvert \geq threshold$<br>$sgn(x) = 1 \quad if\ x \geq threshold; \ \ 0 \ \ o.w$	[2, 3]
RC	Rate of change	$\sum_{n=1}^{N-1} f \lvert x_n - x_{n+1} \rvert$<br>$f(x) = 1 \quad if\ x \geq threshold; \ \ 0 \ \ o.w$	[2, 3]
SSC	Slope sign change	$\sum_{n=2}^{N-1} f[(x_n - x_{n-1}) * (x_n - x_{n+1})]$<br>$f(x) = 1 \quad if\ x \geq threshold; \ \ 0 \ \ o.w$	[2, 3]
DR	Data range	$max(x_n) - min(x_n)\ ;\ n = 1...N$	[9]
ENT	Entropy	$-\sum_{b=1}^{numBins} Pr_b log(Pr_b)\ ;\ numBins = 10$	[9]
LOG	Log detector	$e^{\frac{1}{N}\sum_{n=1}^{N} log\lvert x_n \rvert}$	[2]
MAD	Mean absolute deviation	$\frac{1}{N}\sum_{n=1}^{N} \lvert x_n - MN \rvert$	[3]
Q1, Q2, Q3	1st, 2nd, 3rd quartiles		[9]
IQR	Interquartile range	$Q_3 - Q_1$	[9, 10]
CV	Coefficient of variation	SD/MN	[12, 13]
MD	Median		[9]
MOD	Mode		[9]
TKEO	Teager-Kaiser energy operator	$mean(x_n^2 - (x_{n-1} * x_{n+1}))\ ;\ n = 1...N$	[14, 9]
AR	Auto-regressive coefficients	$x_i = \sum_{p=1}^{P} a_p x_{i-p} + w_i$<br>$P = 4\ ,\ a_i$: AR coefficient , w_i : noise term	[2, 3]

DFA	Detrended fluctuation analysis	$y_k = \sum_{t=1}^{k} x_n - MN$; $k = 1...N$<br>$F_{s,t} = \sqrt{\frac{1}{N}\sum_{k=1}^{N}(y_k - y_{k,s})^2}$;<br>$s = 1...N_t$, $N_t = N/t$,<br>t : num of nonoverlapping segments ,<br>$y_{k,s}$: fitting polynomial in segment s<br>$DFA = F(t) = \left[\frac{1}{2N_t}\sum_{s=1}^{2N_t} F_{s,t}^2\right]^{1/2}$	[15, 9, 11]
HFD	Higuchi's fractal dimension	$X_k^n = X(n+ik)$; $i \in [0, [(N-n)/k]]$,<br>$n \in [1, k]$, n : initial time , k : time interval<br>$L_n(k) = \frac{\left(\sum_{i=1}^{\left[\frac{N-n}{k}\right]}\left\|X(n+ik)-X(n+(i-1)k)\right\|\right)\frac{N-1}{\left[\frac{N-n}{k}\right]k}}{k}$<br>$L_n(k)$: length of the curve X_k^n<br>$\langle L(k)\rangle \propto k^{-D}$<br>$\langle L(k)\rangle$: length of the curve for time interval k<br>$= mean(L_n(k))$ over k sets ,<br>D: fractal dimension , [ ]: Gauss' notation	[11, 16]
KATZ	Katz's fractal dimension	$\frac{log(L/a)}{log(d/a)}$<br>$L$: $\sum$(distances b/w successive points)<br>a : avg(distances b/w successive points)<br>d : max(distances b/w first and other points)	[17]
MAV1	Modified mean absolute value type 1	$\frac{1}{N}\sum_{n=1}^{N} w_i\|x_n\|$<br>$w_i = 1$ if $0.25N \le i \le 0.75N$; 0.5 $o.w$	[2]
MAV2	Modified mean absolute value type 2	$\frac{1}{N}\sum_{n=1}^{N} w_i\|x_n\|$<br>$w_i = 1$ if $0.25N \le i \le 0.75N$;<br>$4i/N$ if $i < 0.25N$; $4(i-N)/N$ $o.w$	[2]
MAVS	Mean absolute value slope	$MAV_{k+1} - MAV_k$; $k = 1...K-1$, $K = 2$	[2]
MBV	Mean binarized values	$\frac{1}{N}\sum_{n=1}^{N} f(x)$<br>$f(x) = 1$ if $x \ge threshold$; 0 $o.w$	[2]
TM4	Absolute temporal moment	$\frac{1}{N}\sum_{n=1}^{N} x^4$	[2]

VFD	Variation fractal dimension	$\widehat{V}_p(l/N) = \frac{1}{2(N-l)} \sum_{i=l}^{N} \lvert X_{i/N} - X_{(i-l)/N} \rvert^p$, $\widehat{V}_p$ = moment estimator of order p ; $l = 1, 2, ..$ $\widehat{D}_{V;p} = 2 - \frac{1}{p} \frac{log(\widehat{V}_p(2/N) - log(\widehat{V}_p(1/N)}{log2}$ $\widehat{D}_{V;p}$: estimator for the fractal dimension	[18]
MAX	Maximum	$max(x_n)$; $n = 1..N$	[19]
MIN	Minimum	$min(x_n)$; $n = 1..N$	[19]
GMN	Geometric mean	$\left(\prod_{n=1}^{N} x_n\right)^{1/N}$	–
HMN	Harmonic mean	$N / \sum_{n=1}^{N} (1/x_n)$	[20, 21]
MDAD	Median absolute deviation	$median(\lvert x_n - MD \rvert)$	[19]

Table S3. Cross-time domain features.

Name	Description	Mathematical equation	References
ENT	Cross-entropy	$H(f,g) = -\int_t f(t)\ \ log(g(t))\ dt$	
CCP	Cross-correlation	$(f \star g)(t) = \int_{-\infty}^{\infty} \overline{f}(\tau)\ \ g(t+\tau)\ d\tau$ $\overline{f}(\tau)$ is the complex conjugate of $f(\tau)$	[22]
MI	Mutual information	$MI(f;g) = \int_t \int_t P_{(f,g)} log\left(\frac{P_{(f,g)}}{P_f P_g}\right) dt\ dt$ $P_{(f,g)}$: joint probability mass function of f and g P_f, P_g : marginal probability mass functions of f and g	[23]

Table S4. The count of misclassified participants with Parkinson's disease (PD) that closely resemble each type of non-PD parkinsonism. UnspecPksm: unspecified parkinsonism.

MSA	Unspec Pksm	PSP	DLB	CBS	ET	DIP
13	6	21	8	11	9	2

Table S5. Confusion matrices of the classifiers using Parkinson's disease (PD) and non-PD parkinsonism (Pksm) participants using features from TUG mobility task. Rows represent actual class and columns represent predictions.

(a) Unsupervised RF feature selection with balanced accuracy maximization

	PD	Pksm
PD	208	52
Pksm	8	10

(b) Mutual information-based ranking for top feature selection

	PD	Pksm
PD	239	21
Pksm	10	8

(c) F_1 score ranking using decision tree classifier for feature scoring

	PD	Pksm
PD	225	35
Pksm	9	9

(d) Supervised RF feature selection with AIC minimization

	PD	Pksm
PD	242	18
Pksm	16	2

(e) Sampling before feature selection with final model trained on original data

	PD	Pksm
PD	250	10
Pksm	15	3

(f) Sampling before feature selection with final model trained on sampled data

	PD	Pksm
PD	258	2
Pksm	18	0

(g) Feature selection before sampling with final model trained on sampled data

	PD	Pksm
PD	251	9
Pksm	14	4

Supplementary Results

We used the RF proximity matrix to determine, for each misclassified PD participant, the type of non-PD parkinsonism they are most like. This matrix gauges similarity between two observations (participants) points by tracking their frequency of ending up at the same terminal node across all trees. When participants exhibit similar features, they frequently traverse the same decision paths. Each time a misclassified PD participant and a participant with non-PD parkinsonism appeared together in the balanced training or testing sets, we used the corresponding proximity matrix of the RF model to calculate their similarity. We then averaged these similarity scores to represent the final similarity between the two participants. This allowed us to calculate the overall similarity between each misclassified PD participant and all participants with non-PD parkinsonism. Then, we assigned the type of non-PD parkinsonism participant with the highest similarity value to the misclassified PD participant (Table S4).

Supplementary Files

The supplementary material available at `https://doi.org/10.13016/2qhy-961s` contains the following compressed files:

1. **data**: folder with sensor readings derived from 32-foot walk, standing with eyes open, standing with eyes closed, two trials of TUG, and two trials of cogTUG. It has also the demographics and clinical evaluation data in a separate `xlsx` file.

2. **code_notebook**: notebook with the code used to generate the results presented in the manuscript. To view the notebook open the file `index.html` in a web browser or open the file `notebook.pdf`.

3. **rdata**: folder with intermediate R objects.

   `sensor_features.RData`: saves a list of the features table of each task.

   `sensor_features_all_tasks.RData`: saves one table of features for all subjects not missing cogTUG data and tasks. The mean and difference of repeated tasks are also added.

   `train_test_splits_PD_PDism`: saves the training and test splits for the five repeats and three-fold cross-validation framework used to build classifiers distinguishing PD from non-PD participants.

   `PD_PDism_miee_split‘i’_iter_‘j’_30`: saves the five random forest models trained on the five balanced subsets of the MIEE algorithm for replicate ‘i’ and fold ‘j’, using features from all mobility tasks.

   `PD_PDism_miee_split‘i’_iter_‘j’_tug_30`: saves the five random forest models trained on the five balanced subsets of the MIEE algorithm for replicate ‘i’ and fold ‘j’, using features from TUG task.

4. **files**: for each of all-tasks and TUG-only classifiers, five `csv` files with the predictions of the ML model for the five replicates.

5. **README**: file with detailed instructions on how to set up and run the code, as well as any dependencies or requirements.

References

1. Cooley, J.W.; Tukey, J.W. An algorithm for the machine calculation of complex fourier series. *Math. Comput.* **1965**, 19, 297–301.

2. Phinyomark, A.; Phukpattaranont, P.; Limsakul, C. Feature reduction and selection for EMG signal classification. *Expert Syst. Appl.* **2012**, 39(8), 7420–7431.

3. Altin, C.; Er, O. Comparison of different time and frequency domain feature extraction methods on elbow gesture's EMG. *Eur. J. Interdiscip. Stud.* **2016**, 5, 35.

4. Oung, Q.; Hariharan, M.; Lee, H.; Basah, S.; Sarillee, M.; Lee, C. Wearable multimodal sensors for evaluation of patients with Parkinson disease. In *2015 IEEE International Conference on Control System, Computing and Engineering (ICCSCE)* **2015** , pp. 269–274.

5. Sinderby, C.; Lindstrom, L.; Grassino, A.E. Automatic assessment of electromyogram quality. *J. Appl. Physiol.* **1995**, 79, 1803–1815. PMID: 8594044.

6. Pepa, L.; Ciabattoni, L.; Verdini, F.; Capecci, M.; Ceravolo, M. Smartphone based fuzzy logic freezing of gait detection in Parkinson's disease. In *2014 IEEE/ASME 10th International Conference on Mechatronic and Embedded Systems and Applications (MESA)* **2014** , pp. 1–6.

7. Mehta, A.; Vaddadi, S.K.; Sharma, V.; Kala, P. A phase-wise analysis of machine learning based human activity recognition using inertial sensors. In *2020 IEEE 17th India Council International Conference (INDICON)* **2020** , pp. 1–7.

8. Bao, L.; Intille, S.S. Activity recognition from user-annotated acceleration data. In Ferscha, A.; Mattern, F., editors, *Pervasive Computing.* Springer Berlin Heidelberg **2004** , pp. 1–17.

9. Arora, S.; Venkataraman, V.; Donohue, S.; Biglan, K.M.; Dorsey, E.R.; Little, M.A. High accuracy discrimination of parkinson's disease participants from healthy controls using smartphones. In *2014 IEEE International Conference on Acoustics, Speech and Signal Processing (ICASSP)* **2014** , pp. 3641–3644.

10. Aich, S.; Youn, J.; Chakraborty, S.; Pradhan, P.M.; Park, J.H.; Park, S.; Park, J. A supervised machine learning approach to detect the on/off state in Parkinson's disease using wearable based gait signals. *Diagnostics* **2020**, 10, 421.

11. Phinyomark, A.; Phukpattaranont, P.; Limsakul, C. Fractal analysis features for weak and single-channel upper-limb EMG signals. *Expert Syst. Appl.* **2012**, 39(12), 11156–11163.

12. Hasni, H.; Yahya, N.; Asirvadam, V.S.; Jatoi, M.A. Analysis of electromyogram (EMG) for detection of neuromuscular disorders. In *2018 International Conference on Intelligent and Advanced System (ICIAS)* **2018** , pp. 1–6.

13. Sukumar, N.; Taran, S.; Bajaj, V. Physical actions classification of surface EMG signals using VMD. In *2018 International Conference on Communication and Signal Processing (ICCSP)* **2018** , pp. 0705–0709.

14. Kaiser, J. On a simple algorithm to calculate the 'energy' of a signal. In *International Conference on Acoustics, Speech, and Signal Processing* **1990** , pp. 381–384 vol.1.

15. Penzel, T.; Kantelhardt, J.; Grote, L.; Peter, J.; Bunde, A. Comparison of detrended fluctuation analysis and spectral analysis for heart rate variability in sleep and sleep apnea. *IEEE Trans. Biomed. Eng.* **2003**, 50(10), 1143–1151.

16. Higuchi, T. Approach to an irregular time series on the basis of the fractal theory. *Phys. D Nonlinear Phenom.* **1988**, 31, 277–283.

17. Katz, M.J. Fractals and the analysis of waveforms. *Comput. Biol. Med.* **1988**, 18(3), 145–156.

18. Gneiting, T.; Ševčíková, H.; Percival, D.B. Estimators of fractal dimension: assessing the roughness of time series and spatial data. *Stat. Sci.* **2012**, 27(2), 247–277.

19. Quiroz, J.C.; Banerjee, A.; Dascalu, S.M.; Lau, S.L. Feature selection for activity recognition from smartphone accelerometer data. *Intell. Autom. Soft Comput.* **2017** , pp. 1–9.

20. Ayman, A.; Attalah, O.; Shaban, H. An efficient human activity recognition framework based on wearable imu wrist sensors. In *2019 IEEE International Conference on Imaging Systems and Techniques (IST)* **2019** , pp. 1–5.

21. Batool, M.; Jalal, A.; Kim, K. Sensors technologies for human activity analysis based on svm optimized by pso algorithm. In *2019 International Conference on Applied and Engineering Mathematics (ICAEM)* **2019** , pp. 145–150.

22. Venables, W.; Ripley, B. *Modern Applied Statistics with S.* Springer New York New York, NY **2002**.

23. Cover, T.; Thomas, J. *Elements of Information Theory.* John Wiley & Sons, Ltd **1991**.
